# Synthesis, Biodistribution and *In vitro* Evaluation of Brain Permeable High Affinity Type 2 Cannabinoid Receptor Agonists [^11^C]MA2 and [^18^F]MA3

**DOI:** 10.3389/fnins.2016.00431

**Published:** 2016-09-22

**Authors:** Muneer Ahamed, Daisy van Veghel, Christoph Ullmer, Koen Van Laere, Alfons Verbruggen, Guy M. Bormans

**Affiliations:** ^1^Laboratory for Radiopharmacy, KU LeuvenLeuven, Belgium; ^2^Roche Pharma Research and Early Development, Roche Innovation Center Basel, F. Hoffmann-La Roche LtdBasel, Switzerland; ^3^Division of Nuclear Medicine, University Hospital and KU LeuvenLeuven, Belgium

**Keywords:** Type 2 cannabinoid receptor, CB2 agonists, Positron emission tomography, Radiosynthesis

## Abstract

The type 2 cannabinoid receptor (CB2) is a member of the endocannabinoid system and is known for its important role in (neuro)inflammation. A PET-imaging agent that allows *in vivo* visualization of CB2 expression may thus allow quantification of neuroinflammation. In this paper, we report the synthesis, radiosynthesis, biodistribution and *in vitro* evaluation of a carbon-11 ([^11^C]MA2) and a fluorine-18 ([^18^F]MA3) labeled analog of a highly potent *N*-arylamide oxadiazole CB2 agonist (EC_50_ = 0.015 nM). MA2 and MA3 behaved as potent CB2 agonist (EC_50_: 3 nM and 0.1 nM, respectively) and their *in vitro* binding affinity for *h*CB2 was found to be 87 nM and 0.8 nM, respectively. Also MA3 (substituted with a fluoro ethyl group) was found to have higher binding affinity and EC_50_ values when compared to the originally reported trifluoromethyl analog **12**. [^11^C]MA2 and [^18^F]MA3 were successfully synthesized with good radiochemical yield, high radiochemical purity and high specific activity. In mice, both tracers were efficiently cleared from blood and all major organs by the hepatobiliary pathway and importantly these compounds showed high brain uptake. In conclusion, [^11^C]MA2 and [^18^F]MA3 are shown to be high potent CB2 agonists with good brain uptake, these favorable characteristics makes them potential PET probes for *in vivo* imaging of brain CB2 receptors. However, in view of its higher affinity and selectivity, further detailed evaluation of MA3 as a PET tracer for CB2 is warranted.

## Introduction

The two types of cannabinoid receptors, CB1 and CB2, together with their endogenous lipid ligands (endocannabinoids) and all proteins responsible for synthesis, transport and degradation of these endocannabinoids represent the endocannabinoid system (ECS) (Rodríguez de Fonseca et al., 2005; Mackie, 2008). CB1 and CB2 are both G-protein coupled receptors, but exhibit different expression patterns and signaling profiles. CB1 receptors are mainly located in brain and are responsible for the psychoactive effects of cannabinoids (Piomelli, 2003), whereas cannabinoid's immunomodulatory activity is assigned to CB2 receptors, which are predominantly expressed on β-lymphocytes and organs and tissues related to the immune system such as the spleen and lymph nodes (Lynn and Herkenham, 1994; Galiegue et al., 1995).

In addition, low levels of CB2 mRNA and protein have been detected in healthy brain in Purkinje cells of the cerebellum, hippocampal neurons, and various nuclei of the brain stem (Atwood and Mackie, 2010). This may indicate that the physiological role of CB2 in the central nervous system (CNS) has been underestimated and that both CB1 and CB2 may control central functions. CB2 is, however, up-regulated in the CNS under neuroinflammation. This CB2 overexpression has been predominantly assigned to microglia, the resident immune cells of the brain, and alters depending on their activation state(Cabral and Griffin-Thomas, 2009; Atwood and Mackie, 2010). CB2-positive microglia have been detected in β-amyloid plaques of Alzheimer's disease patients (Ramirez et al., 2005), in spinal cords of an amyotrophic lateral sclerosis (ALS) mouse model (Shoemaker et al., 2006), in active plaques of patients with multiple sclerosis (MS) (Benito et al., 2007) and in striatal lesions of Huntington's disease (HD) transgenic mouse models and patients (Palazuelos et al., 2009). Moreover, selective CB2 activation results in a decrease of microglial activation in HD and ALS transgenic mouse models and appears to be effective in reducing neurodegeneration (Shoemaker et al., 2006; Palazuelos et al., 2009; Sagredo et al., 2009). These observations suggest that therapeutic modulation of CB2 may be a new promising treatment for neuropathogenic disorders characterized by a neuroinflammatory component.

Various CB2-selective drugs and companion PET-imaging agents have been developed over the past years (Evens et al., 2008, 2009, 2011; Horti et al., 2010; Turkman et al., 2012; Slavik et al., 2015, 2016; Moldovan et al., 2016). Horti and co-workers demonstrated binding of [^11^C]A-836339 to CB2 in lipopolysaccharide (LPS)-induced neuroinflammation and AD mouse models (Horti et al., 2010). Our group previously showed [^11^C]NE40 had CB2-specific retention in the spleen of mice (Evens et al., 2009) and in a rat model with local overexpression of a human inactive CB2 variant (Evens et al., 2012). Rühl et al. reported the synthesis of a ^18^F-labeled *N*-aryl-oxadiazolyl-propionamide derivative with low nanomolar binding affinity for hCB2 (Rühl et al., 2012). The non-radioactive compound was shown to bind CB2 in mouse spleen in an autoradiographic study using [^3^H]CP55940 as radioligand.

There is still a need for CB2 PET-tracers with improved imaging profiles and high affinity and selectivity for CB2. Such high affinity PET-radioligands may allow *in vivo* visualization of the low density brain CB2 receptors. Moreover, agonists preferentially bind to cannabinoid receptors in their (functionally) activated conformation (Gullapalli et al., 2010). Therefore, agonist CB2 PET-radioligands may allow visualization of the activated fraction of the receptor which may correlate better with CB2 related pathology of the receptor system. Here we report the synthesis, radiosynthesis, and biological evaluation of potent carbon-11 and a fluorine-18 labeled CB2 agonists as potential PET tracers for *in vivo* imaging of brain CB2 receptors.

## Results

### Synthesis and radiolabelling

**Figure 1:**
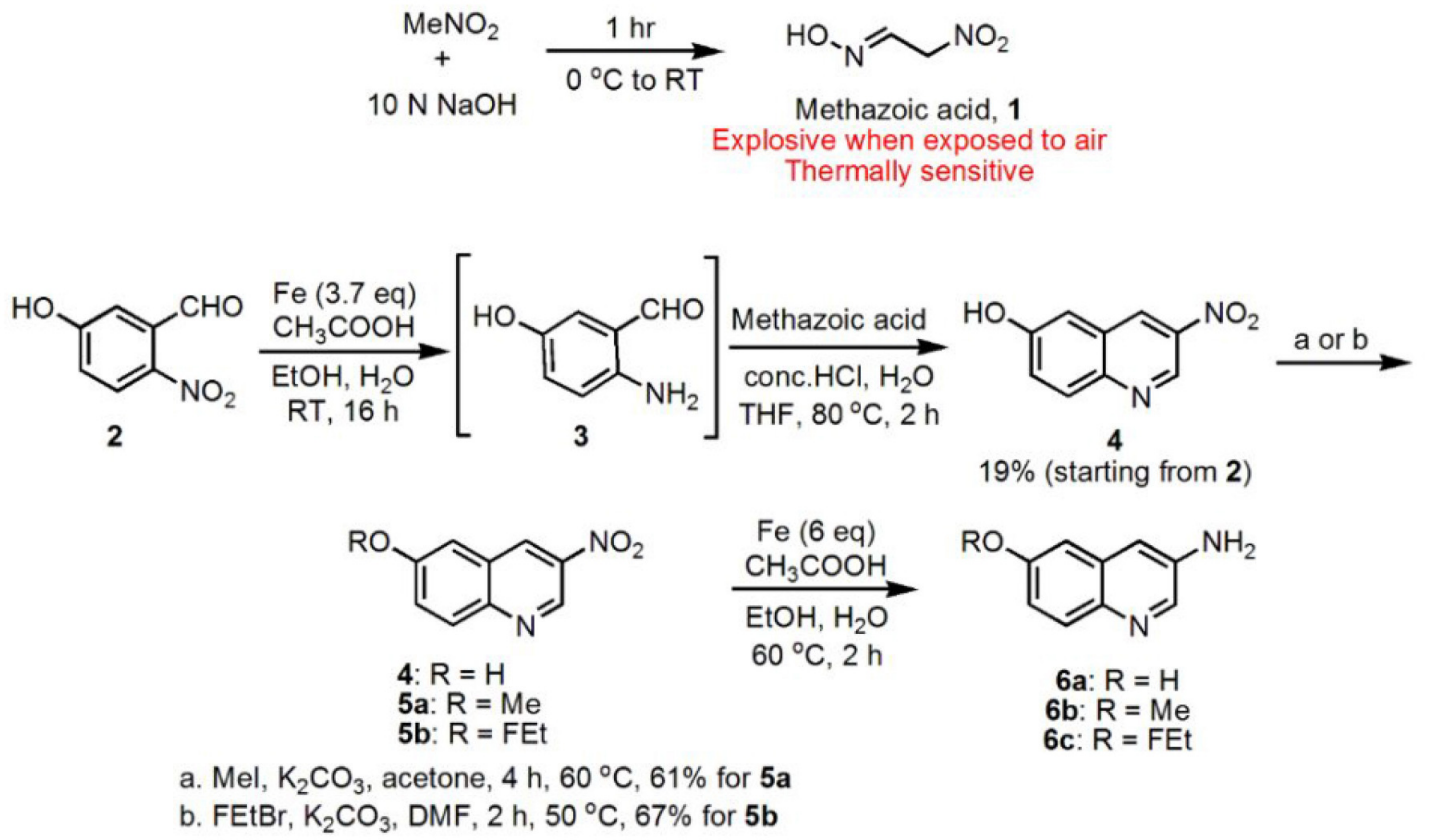
**Synthesis of substituted 3-amino quinolines (6a-c)**.

Compound **1** was synthesized using a previously reported procedure where nitromethane (MeNO_2_) was treated with 10 M sodium hydroxide (NaOH) to form potentially explosive methazoic acid **1**. (Cheng et al., 2008; DiMauro et al., 2008) Condensation of **1** with *in situ* generated *ortho*-aminoaldehyde (**3**) formed 3-nitro-6-hydroxyquinoline **4**, in 19% overall yield starting from compound **2**. Compound **4** was methylated using methyl iodide (MeI) in the presence of potassium carbonate (K_2_CO_3_) under reflux to give 3-nitro-6-methoxyquinoline **5a** in 61% yield. Also compound **4** was converted into **5b** in 67% yield, using 1-Bromo-2-fluoroethane (FEtBr) and K_2_CO_3_ in DMF. Finally, **6**, **6a**, and **6b** were synthesized (36, 45, and 60% yields respectively) by reducing the corresponding nitro compounds **4**, **5a**, and **5b** respectively, *via* a standard reduction using iron (Fe) powder/acetic acid (Figure 1).

**Figure 2:**
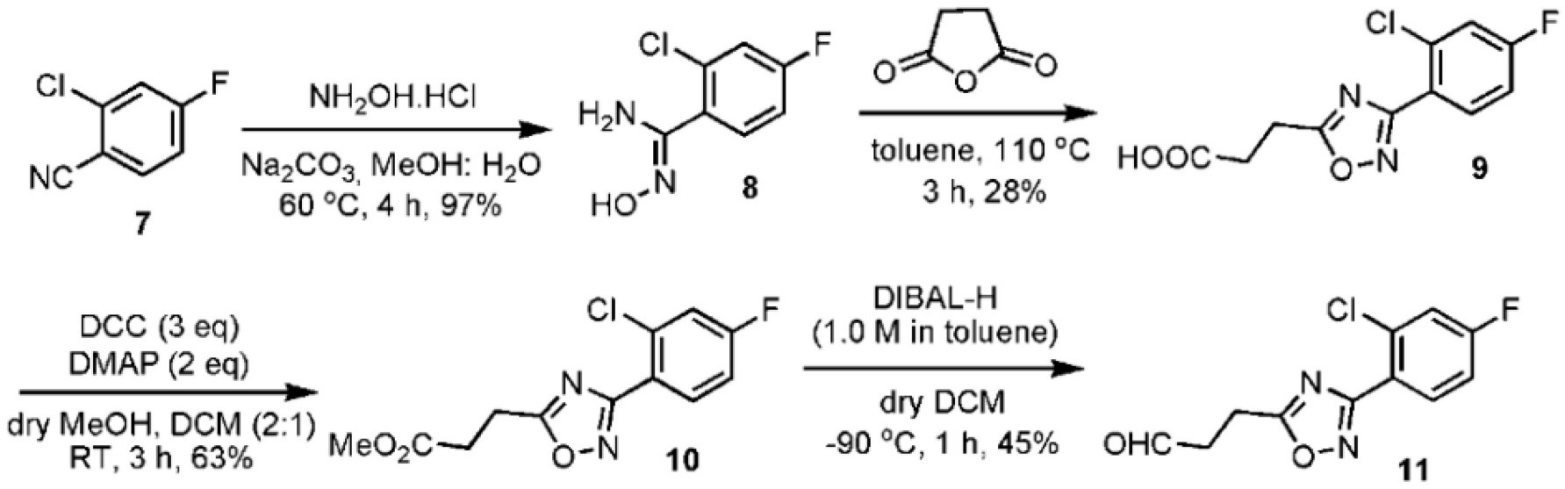
**Synthesis of the oxadiazole fragment 11**.

The oxadiazole fragment (**11**) was synthesized as shown in Figure 2. Commercially available 2-chloro-4-fluoro-benzonitrile (**7**) was converted into an oxime (**8**) in 97% yield when treated with hydroxylamine hydrochloride (NH_2_OH·HCl). The oxadiazole core was formed by condensing **8** with succinic anhydride and obtained in 28% yield after column purification. The carboxylic acid (**9**) then underwent a Steglich esterification (Neises and Steglich, 1978) with *N,N*′-dicyclohexylcarbodiimide (DCC)/4-dimethylaminopyridine (DMAP) to form the desired methyl ester (**10**) in 63% yield. The ester group of compound **10** was then subjected to diisobutylaluminium hydride (DIBAL-H) reduction at −80°C to form the corresponding aldehyde (**11**) in 45% yield. Longer reaction times and increasing DIBAL-H loading led to the formation of a side product, presumably the corresponding alcohol.

**Figure 3:**
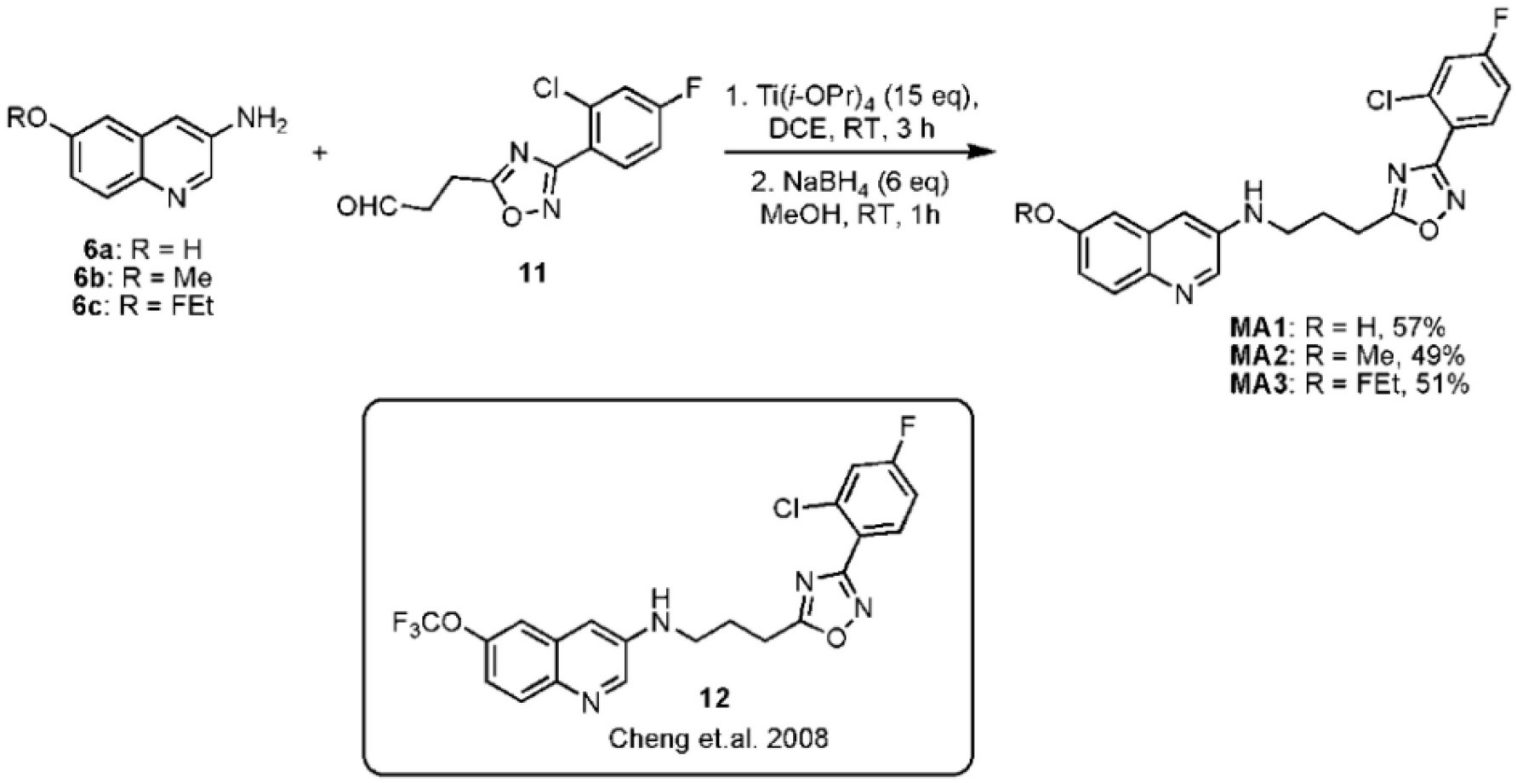
**Final step to synthesize precursor MA1 and reference compounds MA2 and MA3 with structure of compound 12**.

The final reductive amination of **11** with **6a-c** was performed using an excess of titanium isopropoxide (Ti(O-*i*Pr)_4_), followed by reduction with sodium borohydride (NaBH_4_) to form respectively, the desired precursor 3-(3-(3-(2-chloro-4-fluorophenyl)-1,2,4-oxadiazol-5- yl)propylamino)quinolin-6-ol (MA1, 57% yield) and reference compounds *N*-(3-(3-(2- chloro-4-fluorophenyl)-1,2,4-oxadiazol-5-yl)propyl)-6-methoxyquinolin-3-amine (MA2, 49% yield), 6-(2-Fluoroethoxy)-*N*-(3-(3-(2-chloro-4-fluorophenyl)-1,2,4-oxadiazol-5-yl) propyl)quinolin-3-amine (MA3, 51% yield) as shown in Figure 3. Compound **12** was synthesized using a literature procedure and NMR and MS data matched reported data (Cheng et al., 2008).

**Figure 4:**
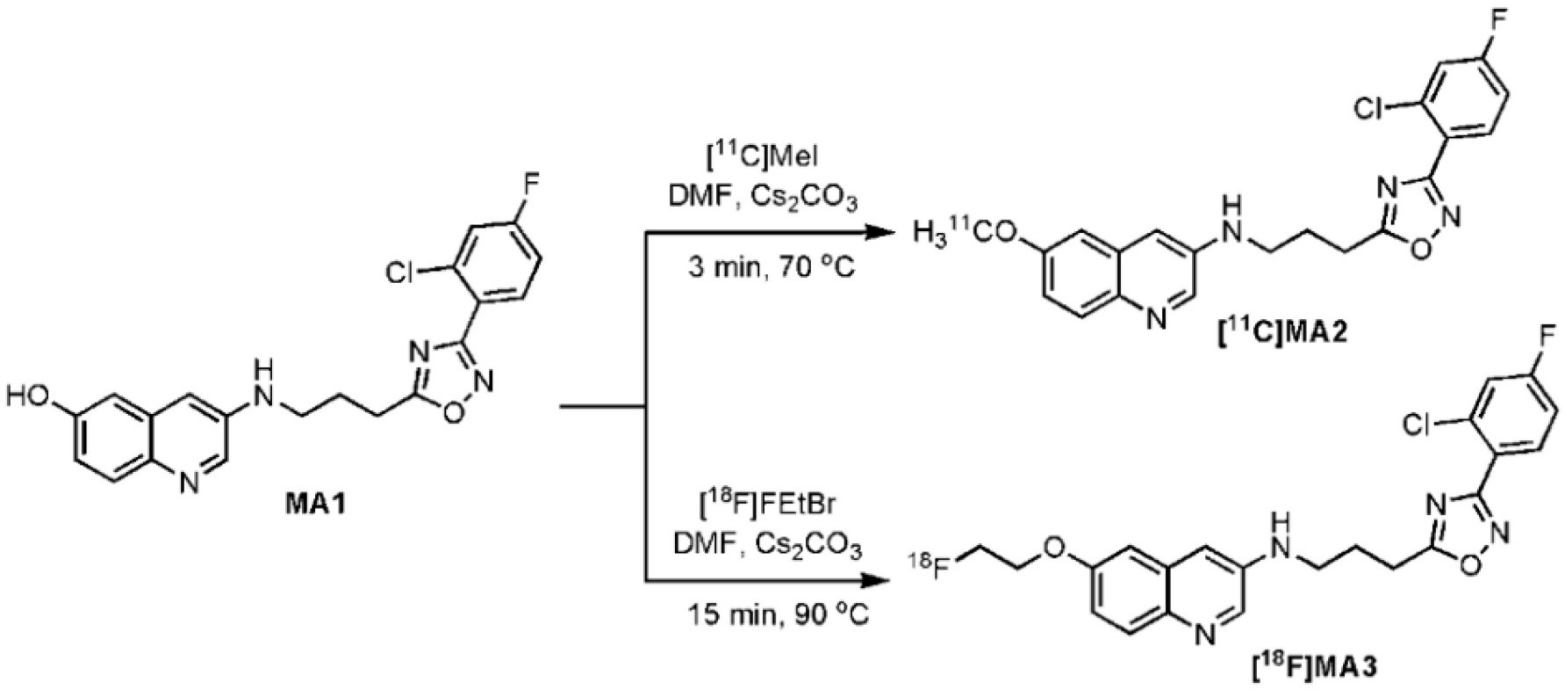
**Radiosynthesis of [^**11**^C]MA2 and [^**18**^F]MA3**.

[^11^C]MA2 and [^18^F]MA3 were successfully produced *via* a nucleophilic substitution reaction on the phenol moiety of precursor MA1 using, respectively, [^11^C]MeI or 1-bromo- 2-[^18^F]fluoroethane ([^18^F]FEtBr) as presented in Figure 4. Carbon-11 methylation yields ranged from 34 to 47% of HPLC-recovered radioactivity relative to [^11^C]MeI, with corresponding isolated amounts of 1628–3145 MBq. Fluorine-18 alkylations yielded isolated amounts of 617–706 MBq (24–68% of HPLC-recovered radioactivity relative to [^18^F]FEtBr). The desired radiolabeled compounds were separated from the precursor, unreacted [^11^C]MeI or [^18^F]FEtBr, and side products by high-performance liquid chromatography (HPLC) yielding over 98% pure [^11^C]MA2 and over 99% pure [^18^F]MA3 with a specific activity of 518 ± 284 GBq/μmol (*n* = 5) and 560 GBq/μmol (*n* = 2), respectively. Non-radioactive MA2 or MA3 were co-injected on the analytical HPLC system to confirm the identity of, respectively, [^11^C]MA2 and [^18^F]MA3.

### Biodistribution studies

The tissue distribution and kinetics of [^11^C]MA2 and [^18^F]MA3 were studied in male NMRI mice at 2, 10, 30, and 60 min post injection of the tracer. The results of the biodistribution studies are presented in Table 1 ([^11^C]MA2) and Table 2 ([^18^F]MA3) as percentage of injected dose (% ID) and standard uptake value (SUV).

**Table 1:** **Tissue distribution of [^**11**^C]MA2 in control mice at 2 and 60 min post injection (***n*** = 4 per time point)**.

**[^11^C]MA2**	**% ID**^a^		**SUV**^b^	
	**2 min**	**60 min**	**2 min**	**60 min**
Bladder + urine	0.2 ± 0.1	4.5 ± 1.0	–	–
Kidneys	8.3 ± 1.6	1.0 ± 0.3	5.1 ± 0.7	0.6 ± 0.1
Liver	28.3 ± 2.9	9.1 ± 3.0	6.0 ± 0.1	2.0 ± 0.5
Spleen	0.5 ± 0.2	0.03 ± 0.01	2.0 ± 0.2	0.2 ± 0.0
Pancreas	1.6 ± 0.8	0.2 ± 0.1	2.2 ± 1.0	0.3 ± 0.0
Lungs	1.4 ± 0.3	0.1 ± 0.0	2.3 ± 0.4	0.2 ± 0.0
Heart	1.4 ± 0.4	0.1 ± 0.0	3.3 ± 0.6	0.1 ± 0.0
Intestines	9.7 ± 0.4	63.8 ± 2.9	–	–
Stomach	1.3 ± 0.3	4.2 ± 3.9	–	–
Brain	1.6 ± 0.5	0.1 ± 0.0	1.8 ± 0.2	0.1 ± 0.0
Blood	5.8 ± 0.4	0.7 ± 0.1	0.8 ± 0.1	0.1 ± 0.0
				

*Mice were anesthetized with isoflurane (2% in oxygen) and injected i.v. with ~9.25 MBq. Data are expressed as mean ± standard deviation*;

a *Percentage of injected dose calculated as counts per minute in organ/total counts per minute recovered*;

b *Standard uptake value calculated as (radioactivity as counts per minute in organ/weight of the organ in grams)/(total counts recovered / body weight in grams)*.

**Table 2:** **Tissue distribution of [^**18**^F]MA3 in control mice at 2, 10, 30, and 60 min post injection (***n*** = 3 or 4 per time point)**.

**[^18^F]MA3**	**% ID**^a^				**SUV**^b^			
	**2 min**	**10 min**	**30 min**	**60 min**	**2 min**	**10 min**	**30 min**	**60 min**
Bladder + urine	0.2 ± 0.2	0.4 ± 0.0	1.7 ± 0.3	1.6 ± 1.4	–	–	–	–
Kidneys	9.0 ± 1.2	1.6 ± 0.2	1.0 ± 0.2	0.7 ± 0.3	5.4 ± 0.4	1.1 ± 0.1	0.6 ± 0.1	0.5 ± 0.1
Liver	31.4 ± 8.4	26.1 ± 3.4	17.3 ± 6.7	10.2 ± 5.2	7.2 ± 2.0	4.7 ± 0.7	3.7 ± 1.4	2.1 ± 1.1
Spleen	0.4 ± 0.1	0.3 ± 0.1	0.1 ± 0.0	0.03 ± 0.01	5.1 ± 0.6	0.5 ± 0.0	0.5 ± 0.3	0.5 ± 0.1
Pancreas	1.4 ± 0.3	0.4 ± 0.1	0.2 ± 0.1	0.1 ± 0.0	3.0 ± 0.5	0.8 ± 0.0	0.4 ± 0.1	0.3 ± 0.1
Lungs	1.2 ± 0.3	0.5 ± 0.0	0.2 ± 0.0	0.1 ± 0.0	2.8 ± 0.7	0.7 ± 0.1	0.4 ± 0.1	0.3 ± 0.1
Heart	1.2 ± 0.1	0.3 ± 0.0	0.1 ± 0.0	0.1 ± 0.0	3.4 ± 0.3	0.6 ± 0.0	0.4 ± 0.1	0.3 ± 0.0
Intestines	9.4 ± 3.9	30.8 ± 6.1	56.2 ± 5.4	64.4 ± 2.6	–	–	–	–
Stomach	1.4 ± 0.2	0.5 ± 0.0	0.8 ± 1.0	4.4 ± 5.1	–	–	–	–
Brain	1.2 ± 0.2	1.0 ± 0.1	0.3 ± 0.1	0.1 ± 0.0	1.1 ± 0.1	0.5 ± 0.0	0.2 ± 0.0	0.1 ± 0.0
Blood	5.4 ± 0.4	2.2 ± 0.3	1.2 ± 0.1	1.0 ± 0.3	0.8 ± 0.1	0.3 ± 0.0	0.2 ± 0.0	0.1 ± 0.0
Bone	5.3 ± 1.2	3.5 ± 0.2	1.6 ± 0.5	2.2 ± 0.3	0.4 ± 0.1	0.3 ± 0.0	0.1 ± 0.0	0.2 ± 0.0

*Mice were anesthetized with isoflurane (2% in oxygen) and injected i.v. with ~0.92 MBq. Data are expressed as mean ± standard deviation*;

a *Percentage of injected dose calculated as counts per minute in organ/total counts per minute recovered*;

b *Standard uptake value calculated as (radioactivity as counts per minute in organ/weight of the organ in grams)/(total counts recovered/body weight in grams)*.

[^11^C]MA2 and [^18^F]MA3 were efficiently cleared from blood (% ID 2 min/60 min ratio = 8.6 and 5.2, respectively) and all major organs. Elimination of the tracers occurred predominantly *via* the hepatobiliary pathway with excretion of radioactivity (*via* liver) into the intestines ([^11^C]MA2: 63.8% ID and [^18^F]MA3: 64.4% ID at 60 min post injection) and to a lesser extent *via* renal pathway, as urinary excretion was minimal with only 4.5% ID ([^11^C]MA2) and 1.6% ID ([^18^F]MA3) at 60 min after injection of the tracers. This is expected, as usually compounds with higher lipophilicity are expected to be excreted *via* hepatobiliary pathway. The calculated logD and polar surface area (PSA) values for [^11^C]MA2 [logD = 4.7; PSA = 73 Å (Mackie, 2008)] and [^18^F]MA3 [clogD = 4.9; PSA = 73 Å (Mackie, 2008)]suggest that the tracers may cross the blood-brain barrier (BBB) through passive diffusion. In accordance, brain uptake of [^11^C]MA2 (1.6% ID at 2 min post injection) was higher than brain uptake of [^18^F]MA3 (1.2% ID at 2 min post injection) although the difference was not statistically significant (*p* = 0.7), but was followed by a rapid wash-out from brain (% ID 2 min/60 min ratio = 18.4 and 9.2, respectively). None of the studied organs, except the liver (SUV = 2.0 and 2.1, respectively, at 60 min post injection), retained [^11^C]MA2 or [^18^F]MA3.

### Radiometabolites

The fraction of radiometabolites in mouse plasma at 2 and 30 min after injection of the tracer was determined by RP-HPLC analysis to investigate the *in vivo* stability of [^11^C]MA2. The obtained data show that [^11^C]MA2 is rapidly metabolized *in vivo*. At 2 min post injection of [^11^C]MA2, 88% (*n* = 2) of the recovered radioactivity was assigned to the intact parent tracer. The percentage of intact [^11^C]MA2 decreased to 34% at 30 min post injection of the tracer. All detected radiometabolites were more polar than the intact tracer.

### Binding profile

The results from the binding and functional assays are presented in Table 3. MA2, MA3, and **12** behaved as agonists with an EC_50_ of, respectively, 3, 0.13, and 0.15 nM for human CB2 (hCB2) and an efficacy of 101, 102, and 100% in the cAMP assays. Among the tested compounds MA3 exhibited the highest binding affinity (K_i_) for hCB2 (K_i_ = 0.8 nM) and had about 4 times higher affinity compared to **12**, about 100 times higher affinity compared to MA2 and 5 times higher affinity compared to NE40. MA2 showed a lower selectivity toward the CB1 receptor (K_i_ ratio hCB1/hCB2 = 19) compared with MA3 (K_i_ ratio hCB1/hCB2 = 127), NE40 (K_i_ ratio hCB1/hCB2 = 241), and 12 (K_i_ ratio hCB1/hCB2 = 202). NE40 behaved as an inverse agonist in the cAMP assay displaying an EC_50_ of 8 nM for hCB2 and a negative efficacy (−178%). With NE40 no species difference in binding affinity was observed.

**Table 3:** **Radiometabolite analysis in blood plasma**.

**% of intact tracer in blood plasma (average of 2 assays)**	**2 min**	**30 min**
[^11^C]MA2	88	34

## Discussion

The aim of this study was to synthesize new high-affinity CB2 agonists, evaluate their brain uptake and potential for *in vivo* visualization of peripheral and brain CB2 receptors. A highly potent *N*-arylamide oxadiazole CB2 agonist was selected from literature as lead compound for the development of our radiolabeled analog 25. However, it should be noted that in the paper of Cheng et al. (2008) the position of the nitrogen atom in the biaryl moiety of the lead compound's structure is incorrectly indicated. A CB2 agonist PET-radioligand would allow to study the functional state of the CB2 receptor system under (patho)physiological conditions. The tracers were obtained in favorable yields, with a high radiochemical purity and high specific activity.

Though there may be some exceptions, the optimal logD value for molecules to cross the BBB *via* passive diffusion is assumed to be between 2.0 and 3.5 (Pike, 2009) and the PSA value is ideally less than 90 Å (van de Waterbeemd et al., 1998). As shown in Table 4, replacement of trifluoromethyl group (**12**) with fluoroethyl group (MA3) leads to higher Ki and EC_50_ values among the tested compounds both in mouse and human varients whereas substitution of the trifluoromethyl with a methyl group (MA2) decreases affinity. Despite slightly higher lipophilicity for MA2 compared to MA3 (clogD = 4.7 and 4.9 respectively), [^11^C]MA2 have crossed the BBB efficiently (1.6 and 1.2% of ID at 2 min post injection respectively) and wash-out from brain was rapid for both the tracers. The brain uptake of [^11^C]MA2 was found to be similar to that of [^11^C]NE40 (% ID = 1.7 in mouse brain at 2 min post injection) (Evens et al., 2012) and in comparision to that of [^11^C]A-836339 (SUV ~ 0.5 in rat brain at 2 min post injection) (Horti et al., 2010). The biodistribution studies also showed that [^11^C]MA2 and [^18^F]MA3 were cleared from blood *via* the hepatobiliary system, as could be expected for compounds with lipophilic properties. [^11^C]MA2 was substantially metabolized as a significant fraction (> 60%) of unidentified polar radiometabolites was found in plasma of mice at 30 min after injection of the tracer. Besides the activity in the liver, due to metabolism and/or excretion of the tracers, radioactivity in other major organs was negligible at 60 min after injection of [^11^C]MA2 or [^18^F]MA3. Retention of the tracers in the spleen was anticipated, as this organ is known to express high levels of CB2 (Lynn and Herkenham, 1994; Galiegue et al., 1995). Surprisingly no accumulation of [^11^C]MA2 or [^18^F]MA3 was observed in the spleen and tracer washout from the spleen was comparable to that from other organs such as the pancreas and lungs. Previously, a lack of retention in the spleen of mice and rats was also observed for other specific CB2 PET-tracers including [^11^C]GW405833 (Evens et al., 2011), [^18^F]FE-GW405833 (Evens et al., 2011), and a ^18^F-labeled 2-oxoquinoline derivative (Turkman et al., 2012). The low spleen uptake could also due to the lower binding affinity of [^11^C]GW405833 (35 nM) and [^18^F]FE-GW405833 (27 nM) for CB2 or in the case of the ^18^F-labeled 2-oxoquinoline derivative a low specific activity (44.4 GBq/μmol) (Turkman et al., 2012). Also recently, a 1,3,5-triazine based CB2 agonist labeled with fluorine-18 was reported to have a low spleen uptake (0.39% ID/g, at 15 min) (Yrjölä et al., 2015). In general agonists bind to activated receptor sites, which only represent a fraction of total binding sites, and this may explain the low spleen uptake. However, the specific *in vivo* binding of these tracers to CB2 receptors has to be established, experiments with these two tracers on local overexpression of *h*CB2 in rats (as previously shown) (Evens et al., 2011; Turkman et al., 2012) are currently underway.

**Table 4:** **Characterization of MA2, MA3, 12, and NE40 in functional cAMP assays and radioligand binding assays with [^**3**^H]CP55940**.

	**NE40**	**MA2**	**MA3**	**12**
*h*CB2 cAMP EC_50_ [nM] (% eff)	8 (−178)	3 (101)	0.13 (102)	0.15 (100)
*m*CB2 cAMP EC_50_ [nM] (% eff)	11 (−128)	2 (100)	0.09 (101)	0.18 (100)
EC_50_ ratio *h*CB1/*h*CB2	> 1250	430	563	1300
*h*CB2 K_i_ [nM]	4	87	0.8	2.8
*h*CB1 K_i_ [nM]	1037	1611	102	568
K_i_ ratio *h*CB1/*h*CB2	241	19	127	202
*m*CB2 K_i_ [nM]	2	241	2.6	4.6

## Conclusions

We successfully synthesized MA2 and MA3 and their *in vitro* binding affinity for *h*CB2 was found to be 87 nM and 0.8 nM, respectively. Radiolabeled [^11^C]MA2 and [^18^F]MA3 was obtained in relatively high yields, high radiochemical purity, and high specific activity. Preliminary biodistribution studies indicate favorable brain uptake and clearance from blood and major organs. These preliminary results indicate [^11^C]MA2 and [^18^F]MA3 can be potential tracers for imaging brain CB2 receptors, in the view of their higher CB2 affinity and good brain uptake. It has to be noted that, not many CB2 agonists are available for pharmacologic studies that have both high affinity and excellent brain uptake and the compounds described here are suited to perform pharmacological experiments studying the role of CB2 in CNS.

## Materials and methods

### General

2-Chloro-4-fluorobenzonitrile was purchased from TCI Europe (Zwijndrecht, Belgium). All other chemicals and solvents were obtained from Acros Organics (Geel, Belgium), Sigma-Aldrich (Bornem, Belgium), or VWR International (Leuven, Belgium). For ascending thin layer chromatography (TLC), pre-coated aluminum backed plates (Silica gel 60 with fluorescent indicator UV 254 nm, 0.2 mm thickness; Macherey-Nagel, Düren, Germany) were used. Molecular mass measurements were performed on a time-of-flight mass spectrometer (LCT, Micromass, Manchester, UK) equipped with an orthogonal electrospray ionization (ESI) interface. Acquisition and processing of data was done using MassLynx software (version 3.5, Micromass). Mass data were rounded to the whole number. ^1^H nuclear magnetic resonance (NMR) spectra were acquired with a Bruker Avance II spectrometer (400 MHz, 5 mm probe, Fällanden, Switzerland). Chemical shifts are reported in parts per million relative to tetramethylsilane (δ = 0). Coupling constants are reported in hertz (Hz). Splitting patterns are defined by s (singlet), d (doublet), t (triplet), or m (multiplet). The clogD and PSA values were calculated using, respectively, Marvinsketch 5.7.0 software and the calculator from Daylight (http://www.daylight.com/meetings/emug00/Ertl/tpsa.html). HPLC analysis was performed on an L2130 LaChrom Elite pump (Hitachi, Tokyo, Japan) connected to a UV L2400 LaChrom Elite spectrometer (Hitachi). Radiolabeled compounds were analyzed by passage of the HPLC eluate (after passing through the UV detector) over a 3-inch NaI(Tl) scintillation detector connected to a single channel analyzer (GABI box; Raytest, Straubenhardt, Germany). Data were acquired and analyzed using a RaChel (Lablogic, Sheffield, UK) or GINA Star (Raytest) data acquisition system. Quantification of radioactivity during biodistribution and metabolite studies was performed using an automated gamma counter equipped with a 3-inch NaI(Tl) well crystal coupled to a multichannel analyzer, mounted in a sample changer [2480 Wizard (Mackie, 2008), Perkin Elmer, Massachusetts, USA]. The values are corrected for background radiation, physical decay and counter dead time.

Animals were housed in individually ventilated cages in a thermo-regulated (22°C), humidity-controlled facility under a 12 h light/12 h dark cycle, with free access to food and water. All animal experiments were performed in compliance with the principles set by the Belgian law relating to the conduct of animal experimentation, after approval from the university (KU Leuven) animal ethics committee.

### Organic synthesis

#### Methazoic acid (1)

##### Caution:

*Methazoic acid is a potential explosive, heat, and shock sensitive compound*. To a solution of 10 M NaOH (3 mL) at 0°C was added dropwise MeNO_2_ (0.5 mL) and the resulting mixture was stirred for 15 min. The reaction mixture was allowed to warm to room temperature, stirred for 15 min and a second amount of MeNO_2_ (0.5 mL) was added. After stirring until a clear brown solution was formed (~30 min), ice-cold water (50 mL), and concentrated hydrochloric acid (~11.6 M HCl, 3 mL) were slowly added to the mixture. The resultant solution was extracted with diethyl ether (Et_2_O; 2 × 100 mL) and the organic layers were collected and dried over magnesium sulfate (MgSO_4_). The solvent was carefully removed under reduced pressure without heating to yield compound **1** (~1 g) as an orange-yellow viscous oil, which was used in the synthesis of **4**, without any further purification.

#### 3-nitro-6-hydroxyquinoline (4)

##### Step A:

To a solution of 2-nitro-5-hydroxybenzaldehyde (**2**; 1.20 g, 7.18 mmol, 1.0 eq) in ethanol (EtOH; 30 mL) was added Fe powder (1.48 g, 26.57 mmol, 3.7 eq), followed by acetic acid (2.4 mL) and H_2_O (0.8 mL). After stirring at room temperature for 16 h, the dark brown reaction mixture was diluted with EtOH (30 mL), filtered through a sintered funnel, and concentrated *in vacuo*. The crude mixture was extracted with dichloromethane (DCM; 2 × 200 mL), the combined organic layers were dried over MgSO_4_ and concentrated *in vacuo*. Compound **3** was obtained as a crude yellow powder, which was used in step B without any further purification.

##### Step B:

To a stirred solution of **3** in either tetrahydrofuran (THF) or EtOH (10 mL) was added a solution of compound **1** (~1 g) in a mixture of H_2_O (5 mL) and 5 M HCl (5 mL). The dark brown mixture was heated to 80^*o*^C and stirred for 2 h. The solvent was evaporated under reduced pressure, the crude material was extracted with DCM (3 × 100 mL) and the solution was again concentrated under reduced pressure and purified over silica column to give **4** as a pale brown solid. ^1^H NMR (CDCl_3_): δ 7.10– 7.17 (m, 1H, Ar), 7.41–7.45 (m, 1H, Ar), 8.11 (s, 1H, Ar), 8.67 (s, 1H, Ar), 9.24 (s, 1H, Ar). MS (ESI) m/z: 191.2 [(M+H)^+^, 100%].

#### 3-nitro-6-methoxyquinoline (5a)

To a solution of compound **4** (260 mg, 1.37 mmol, 1 eq) in acetone (15 mL), K_2_CO_3_ (283 mg, 2.05 mmol, 1.5 eq) and MeI (5 eq) were added and the reaction mixture was stirred at 60 °C for 4 h. Completion of reaction was confirmed by TLC (EtOAc/heptane 1:1). Acetone was removed under reduced pressure and the crude residue was diluted with DCM (100 mL) and washed with H_2_O (3 × 25 mL). The organic layer was dried over MgSO_4_ and the solvent was removed under reduced pressure. Silica gel column purification yielded **5** (171 mg) as pale yellow crystalline needles. Yield: 61%. TLC (EtOAc/heptane 1:1): R_f_ = 0.55. ^1^H NMR (CDCl_3_): δ 3.94 (s, 3H, OMe), 7.19–7.23 (m, 1H, Ar), 7.50–7.55 (m, 1H, Ar), 8.05 (s, 1H, Ar), 8.86 (s, 1H, Ar), 9.44 (s, 1H, Ar). MS (ESI) m/z: 205.2 [(M+H)^+^, 100%].

#### 6-(2-fluoroethoxy)-3-nitroquinoline (5b)

To a solution of compound **4** (260 mg, 1.37 mmol, 1 eq) in DMF (15 mL), K_2_CO_3_ (283 mg, 2.05 mmol, 1.5 eq) and FEtBr (5 eq) were added and the reaction mixture was stirred at 50°C for 2 h. Completion of reaction was confirmed by TLC (EtOAc/heptane 1:1). The crude residue was diluted with DCM (100 mL) and washed with H_2_O (3 × 25 mL). The organic layer was dried over MgSO_4_ and the solvent was removed under reduced pressure. Silica gel column purification yielded **5b** (230 mg) as pale yellow crystalline needles. Yield: 67%. TLC (EtOAc/heptane 1:1): R_f_ = 0.60. ^1^H NMR (CDCl_3_): δ 4.36 (dt, 2H, *J* 2.4 & 19.4), 4.84 (dt, 2H, *J* 2.4 & 39.2), 7.22 (s, 1H, Ar), 7.59 (d, 1H, *J* 9.2, Ar), 8.10 (d, 1H, *J* 9.2, Ar), 8.87 (s, 1H, Ar), 9.47 (s, 1H, Ar). MS (ESI) m/z: 236.8 [(M+H)^+^, 100%].

#### 3-amino-6-quinolinols (6a-c)

*3-Aminoquinolin-6-ol (****6a****)* To a solution of compound **4** (330 mg, 1.74 mmol, 1 eq) in EtOH (10 mL), Fe powder (485 mg, 8.68 mmol, 5 eq) was added and the mixture was stirred. After adding acetic acid (1 mL) and H_2_O (0.2 mL), the reaction mixture was heated to 60^*o*^C and stirred for 3 h. The Fe powder was removed by filtering the reaction mixture over a sintered funnel. The filtrate was collected, dried over MgSO_4_ and concentrated to yield **6a** (100 mg) as a pale yellow powder. Yield: 36%. TLC (DCM/MeOH, 95:5): R_f_ = 0.21. MS (ESI) m/z: 161.0 [(M+H)^+^, 100%].

*6-Methoxyquinolin-3-amine (****6b****)* Compound **6b** was synthesized starting from compound **5a** in accordance with the procedure described for compound **6a**. Yellow solid was obtained in 45% yield. TLC (DCM/MeOH 95:5): R_f_ = 0.25. MS (ESI) m/z: 174.7 [(M+H)^+^, 100%].

*6-(2-Fluoroethoxy)quinolin-3-amine (****6c****)* Compound **6c** was also synthesized starting from compound **5b** as described for compound **6a**. Colorless powder was obtained in 60% yield (175 mg). *R*_F_ 0.25 (9.5:0.5 DCM:MeOH). ^1^H NMR (MeOD): δ 4.55 (dt, 2H, *J* 3.5 & 28.8), 5.01 (dt, 2H, *J* 3.4 & 47.8), 7.35 (d, 1H, *J* 9.2, Ar), 7.25 (s, 1H, Ar), 7.51 (s, 1H, Ar), 7.98 (d, 1H, *J* 9.2, Ar) 8.54 (s, 1H, Ar). MS (ESI) m/z: 206.9 [(M+H)^+^, 100%].

#### 2-chloro-4-fluoro-n′-hydroxybenzamidine (8)

To a solution of 2-chloro-4-fluoro-benzonitrile (**7**; 1.0 g, 6.45 mmol, 1.0 eq) in MeOH was added NH_2_OH·HCl (0.58 g, 8.36 mmol, 1.3 eq), Na_2_CO_3_ (1.0 g, 9.64 mmol, 1.5 eq) and H_2_O (4 mL), and the reaction mixture was stirred for 4 h at 60°C. The solvent was evaporated under reduced pressure and the crude residue was extracted with DCM (2 × 500 mL). The combined organic layers were dried over MgSO_4_ and the solvent was removed under reduced pressure. The crude compound **8** (1.18 g, 97%) was used as such in the next step without any further purification. *R*_F_ 0.41 (1:1 hexane:EtOH). MS (ESI) *m/z*: 189.0 [(M+H)^+^, 100%].

#### 3-(3-(2-chloro-4-fluorophenyl)-1,2,4-oxadiazol-5-yl)propanoic acid (9)

A solution of compound **8** (1.0 g, 5.32 mmol, 1.0 eq) and succinic anhydride (0.53 g, 5.32 mmol, 1.0 eq) in toluene (15 mL) was stirred for 3 h at 110°C. Toluene was evaporated under reduced pressure, the crude residue was extracted with DCM (3 × 250 mL), the combined organic extracts were dried over MgSO_4_ and concentrated under reduced pressure. The crude residue was purified using silica gel column chromatography (EtOAc/heptane gradient 10–30% EtOAc) and compound **9** was obtained as a white solid (425 mg). Yield: 28%. *R*_F_ 0.25 (1:1 hexane:EtOH). ^1^H NMR (DMSO-*d*_6_): δ 2.56 (t, 2H, *J* 6.7, CH2), 2.94 (t, 2H, *J* 6.9, CH2), 7.11–7.18 (m, 1H, Ar), 7.39–7.44 (m, 1H, Ar), 7.65–7.71 (m, 1H, Ar). MS (ESI) *m/z*: 269.29 [(MH^¬^), 100%].

#### Methyl-3-(3-(2-chloro-4-fluorophenyl)-1,2,4-oxadiazol-5-yl)propanoate (10)

Compound **9** (100 mg, 0.370 mmol, 1 eq) was dissolved in dry DCM (4 mL) followed by the addition of DCC (229 mg, 1.11 mmol, 3 eq), DMAP (90 mg, 0.740 mmol, 2 eq), and dry MeOH (0.5 mL). The reaction mixture was stirred for 16 h at room temperature. The reaction mixture was concentrated under reduced pressure, DCM (50 mL) was added and the solution was washed with H_2_O (2 × 20 mL). After drying the organic layer over MgSO_4_, the solvent was removed and the crude product purified through silica gel column chromatography (EtOAc/heptane gradient 0–10% EtOAc) yielded pure compound **10**. *Colorless oil (63% yield, 66 mg)*. *R*_F_ 0.55 (1:1 hexane:EtOH). ^1^H NMR (CDCl_3_): δ 2.54 (t, 2H, *J* 7.3, CH2), 2.89 (t, 2H, *J* 7.3, CH2), 3.33 (s, 3H, CH3), 6.66–6.72 (m, 1H, Ar), 6.85–6.89 (m, 1H, Ar), 7.50–7.55 (m, 1H, Ar). MS (ESI) *m/z*: 285.1 [(MH^+^), 100%].

#### 3-(3-(2-chloro-4-fluorophenyl)-1,2,4-oxadiazol-5-yl)propanal (11)

To a solution of compound **10** (100 mg, 0.352 mmol, 1 eq) in dry DCM (5 mL) cooled to −80°C was added drop-wise over 5 min DIBAL-H 1.5 M in toluene (0.107 mL, 0.528 mmol, 1.5 eq) and the reaction mixture was stirred at the same temperature for 1 h. The reaction was quenched with a few drops of H_2_O, DCM (50 mL) was added and the mixture was washed with H_2_O (2 × 20 mL). The organic layer was dried over MgSO_4_ and the solvent was removed under reduced pressure. The crude residue was purified through silica gel flash chromatography using a mixture of heptane/EtOAc (2:1) to yield the desired aldehyde **11** (40 mg). *Colorless powder* (40 mg, 45%). *R*_F_ 0.40 (1:1 hexane:EtOH). ^1^H NMR (CDCl_3_): δ 2.72 (t, 2H, *J* 6.8, CH2), 2.87 (t, 2H, *J* 6.9, CH2), 6.67–6.70 (m, 1H, Ar), 6.85–6.89 (m, 1H, Ar), 7.54–7.56 (m, 1H, Ar), 9.49 (s. 1H, CHO). MS (ESI) *m/z*: 255.1 [(MH^+^), 100%].

#### N-(3-(3-(2-chloro-4-fluorophenyl)-1,2,4-oxadiazol-5-yl)propyl)-6-methoxyquinolin-3-amine (MA2)

Compound **11** (87.0 mg, 0.343 mmol, 1 eq) and amine **6b** (60.0 mg, 0.343 mmol, 1 eq) were dissolved in dichloroethane (DCE; 3 mL), followed by the addition of Ti(O-*i*Pr)_4_ (1.68 mL, 5.142 mmol, 15 eq) at room temperature. After stirring for 1 h, the reaction mixture was added dropwise to a stirred solution of NaBH_4_ (77.8 mg, 2.057 mmol, 6 eq) in dry MeOH (6 mL) at 0^*o*^C. After the addition was complete the mixture was brought to room temperature and stirred for 2 h. The reaction was quenched with a few drops of 1 M HCl and solvents were evaporated under reduced pressure. The residue was dissolved in DCM (100 mL), the solution was washed with H_2_O (2 × 15 mL) and the organic layer was dried and concentrated by vacuum evaporation. The crude product was purified over silica gel chromatography using mixtures of DCM/MeOH as eluent. Pale yellow oil (70 mg), yield: 49%. TLC (hexane/EtOH 1:1): R_f_ = 0.21. ^1^H NMR (CDCl_3_): δ 2.25–2.30 (m, 2H, H^3b^), 3.10–3.16 (m, 2H, H^3c^), 3.40 (t, 2H, *J* 6.7, H^3a^), 3.87 (s, 3H, OMe), 6.86–6.88 (m, 1H, Ar), 6.94–6.97 (m, 1H, Ar), 7.02–7.11 (m, 2H, Ar), 7.21–7.27 (m, 2H, Ar), 7.78–7.83 (m, 1H, Ar), 7.85–7.91 (m, 1H, Ar), 8.25 (br s, 1H, NH). MS (ESI) *m/z*: 412 [(M+H)^+^, 100%].

#### 3-(3-(3-(2-chloro-4-fluorophenyl)-1,2,4-oxadiazol-5-yl)propylamino)quinolin-6-ol (MA1)

**MA1** was synthesized starting from compound **6a** in accordance with the procedure described for **MA2**. Pale yellow solid (90 mg), yield: 57%. TLC (hexane/EtOH 1:1): R_f_ = 0.20. MS (ESI) *m/z*: 398 [(M+H)^+^, 100%]. ^1^H NMR (CDCl_3_): δ 2.50–2.56 (m, 2H, H^3b^), 3.40–3.46 (m, 2H, H^3c^), 3.60–3.65 (m, 2H, H^3a^), 7.16–7.26 (m, 4H, Ar), 7.42–7.48 (m, 1H, Ar), 7.65–7.70 (m, 1H, Ar), 8.09–8.13 (m, 1H, Ar), 8.24–8.28 (m, 1H, Ar), 8.45 (s, 1H, NH). HRMS (ESI) Calcd. for C_20_H_17_ClFN_4_O_2_ [M+H]^+^: 399.1018. Found: 399.1058.

#### 6-(2-fluoroethoxy)-n-(3-(3-(2-chloro-4-fluorophenyl) -1,2,4-oxadiazol-5-yl)propyl)quinolin-3-amine (MA3)

*Yellow crystalline solid* (55 mg, 51%). *R*_F_ 0.25 (1:1 hexane:EtOH). ^1^H NMR (CDCl_3_): δ 2.20–2.30 (m, 2H), 3.11 (t, 2H, *J* 7.2), 3.37 (t, 2H, *J* 6.8), 4.25 (dt, 2H, *J* 4.2 & 27.7), 4.77 (dt, 2H, *J* 4.1 & 47.4), 6.86 (d, 1H, *J* 2.7, Ar), 6.92 (d, 1H, *J* 2.6, Ar), 7.03–7.11 (m, 2H, Ar), 7.23–7.28 (m, 1H, Ar), 7.78–7.89 (m, 2H, Ar), 8.25 (d, 1H, *J* 2.7, Ar). HRMS (ESI) Calcd. for C_22_H_20_ClF_2_N_4_O_2_ (MH^+^): 445.1237. Found: 445.1276.

### Radiosynthesis

[^11^C]CH_3_I and [^18^F]FEtBr were produced according to methods described by Evens et al. (2008) and Chitneni et al. (2008), respectively. [^11^C]CH_3_I was bubbled with a stream of helium through a solution of the precursor MA1 (100 μg) and cesium carbonate (Cs_2_CO_3_; 1–2 mg) in dimethylformamide (DMF, 100 μL) until the radioactivity in the reaction vial was stabilized. The reaction mixture was heated for 3 min at 70°C, diluted with 1 mL of water for injection and applied onto an HPLC column (Waters XBridge RP-C18, 5 μm, 4.6 × 150 mm) that was eluted with a mixture of EtOH/sodium acetate (NaOAc) buffer 0.05 M pH 5.5 (53:47 V/V) as mobile phase at a flow rate of 1 mL/min. The desired product [^11^C]MA2 eluted after 11 min. [^18^F]FEtBr was distilled with a stream of helium and passed through an ascarite column (6 × 150 mm) in a reaction vial containing MA1 (100 μg) and Cs_2_CO_3_ (1–2 mg) in DMF (100 μL). After heating for 15 min at 90°C, the reaction mixture was diluted with 1 mL of water for injection and injected on an XBridge column (5 μm, 4.6 × 150 mm) which was eluted with a mixture of EtOH/NaOAc 0.05 M pH 5.5 (45:55 V/V) at a flow rate of 1 mL/min. The desired product [^18^F]MA3 was collected after 20 min. Quality control of [^11^C]MA2 and [^18^F]MA3 was performed on an analytical HPLC system consisting of an XBridge column (RP-C18, 3.5 μm, 3.0 × 100 mm; Waters) eluted with a mixture of CH_3_CN/NaOAc buffer 0.05 M pH 5.5 ([^11^C]MA2: 45:55 V/V and [^18^F]MA3: 40:60 V/V) at a flow rate of 0.8 mL/min. UV detection was performed at 254 nm.

### Biodistribution studies

The biodistribution of [^11^C]MA2 and [^18^F]MA3 was studied in normal male Naval Medical Research Institute (NMRI) mice. A solution of HPLC-purified [^11^C]MA2 or [^18^F]MA3 was diluted with saline to obtain an ethanol concentration < 10%. Mice were anesthetized with isoflurane (2% in oxygen) and injected intravenously (i.v.) with [^11^C]MA2 (~ 9.25 MBq) or [^18^F]MA3 (~ 0.92 MBq) via a lateral tail vein. The mice were sacrificed by decapitation at 2, and 60 min post injection (for [^11^C]MA2, *n* = 4) and at 2, 10, 30, or 60 min post injection (for [^18^F]MA3, *n* = 3 or 4 per time point) and dissected. Blood, organs, and other body parts were collected in tared tubes and the radioactivity in each tube was measured using an automated gamma counter. The tubes containing selected organs and blood were weighed. For calculation of total radioactivity in blood, blood mass was assumed to be 7% of the body mass (Fritzberg et al., 1982). Quantitative data are expressed as mean ± standard deviation (SD). Means were compared using an unpaired two-tailed student *t*-test. Values were considered statistically significant for *P* ≤ 0.05.

### Plasma radiometabolite analysis

After i.v. administration of [^11^C]MA2 (~ 9.25 MBq) via a lateral tail vein to anesthetized NMRI mice (isoflurane 2% in oxygen), the mice were decapitated at 2 or 30 min post injection (*n* = 2 per time point). Blood was collected into lithium heparin-containing tubes (4.5-mL lithium heparin PST tubes, BD Vacutainer; BD, Franklin Lakes, New Jersey) and stored on ice. The blood samples were centrifuged for 10 min at 3000 rpm to separate the plasma. The isolated plasma was spiked with authentic non-radioactive MA2 (15 μL of 1 mg/mL solution in CH_3_CN) and analyzed by RP-HPLC on a Chromolith RP C_18_ column (3 mm x 100 mm; Merck) eluted with gradient mixtures of CH_3_CN (A) and 0.05 M NaOAc pH 5.5 (B) (0–5 min: Isocratic 100% B, flow rate of 0.5 mL/min; 5–10 min: Linear gradient 100 B to 10% B, flow rate of 1 mL/min; 10–13 min: Isocratic 10% B, flow rate of 1 mL/min; 13–13.1 min: Linear gradient 10 B to 100% B, flow rate of 0.5 mL/min; 13.1–15 min: Isocratic 100% B, flow rate of 0.5 mL/min). After passing through a UV detector (254 nm) and an in-line 3-inch NaI(Tl) scintillation detector, the HPLC eluate was collected as 0.5- or 1-mL fractions (model 2110 fraction collector, Biorad, Hercules, CA). The radioactivity in all fractions was measured using an automated gamma counter. The recovery of the injected radioactivity from the HPLC apparatus and Chromolith column was 94.9 ± 0.9 % (*n* = 4).

### Functional and binding experiments

The competition binding experiments and functional assays were performed by Roche Healthcare.

#### Cell culture

CHO-K1 beta-arrestin cells (DiscoveRx, Fremont, CA) expressing hCB2, mouse CB2 (mCB2), and human CB1 (hCB1) were cultured in F-12 Nutrient Mixture (HAM) supplemented with 10% fetal bovine serum (FBS), 300 μg/mL hygromycin and 800 μg/mL geneticin (G418). Cells were incubated in a humidified atmosphere at 37°C with 5% CO_2_.

#### cAMP assay

cAMP was measured using cAMP-Nano-TRF detection kit (Roche Diagnostics, Penzberg, Germany). Cells were seeded 17–24 h prior to the experiment 3 × 10^4^ cells per well in a black 96-well plate with flat clear bottom (Corning, Wiesbaden, Germany) in growth medium and incubated in 5% CO_2_ at 37°C in a humidified incubator. The growth medium was exchanged with Krebs Ringer bicarbonate buffer with 1 mmol/L 3-isobutyl-1-methylxanthine (IBMX), 0.1% fatty acid-free bovine serum albumin (BSA) and incubated at 30°C for 60 min. Agonist was added to a final assay volume of 100 μL and the mixture was incubated for 30 min at 30°C. The assay was stopped by the addition of 50 μL 3x lysis reagent and shaken for 2 h at room temperature. The time-resolved energy transfer was measured using an LF502 Nanoscan FLT (IOM, Berlin, Germany), equipped with a laser as excitation source. cAMP content was determined from the function of a standard curve spanning from 10 to 0.13 nmol/L cAMP.

#### Radioligand binding assay

Stably transfected cells were treated for 24 h with 5 mM butyrate in growth medium before harvesting, followed by homogenization in 15 mmol/L Hepes, 0.3 mmol/L EDTA, 1 mmol/L EGTA, 2 mmol/L MgCl_2_, complete EDTA-free protease inhibitor (Roche Applied Science, Rotkreuz, Switzerland), pH 7.4 using a glass potter and centrifugation at 47,800 g at 4°C for 30 min. The pellet was then rehomogenized twice in the same buffer and centrifuged (47,800 g, 4°C, 30 min). The final pellet was then resuspended in 75 mmol/L Tris, 0.3 mmol/L EDTA, 1 mmol/L EGTA, 12.5 mmol/L MgCl_2_, 250 mmol/L sucrose, pH 7.4 at a protein concentration of 1 to 3 mg/mL, aliquoted, frozen on dry ice and stored at −80°C. Saturation binding was performed with 0.05 to 2.6 nM [^3^H]CP55940 (Perkin Elmer) and 1.0 μg of membrane protein. CP55940 (10 μM) was used to define non-specific binding. More than 95% of the total binding signal was specific. Assay buffer consisted of 50 mmol/L Tris-HCl, 5 mmol/L MgCl_2_, 2.5 mmol/L EGTA, and 0.1% fatty acid-free BSA, pH 7.4. Assays were initiated by addition of membranes in a final volume of 250 μL/well. Mixtures were incubated for 3 h at room temperature and then vacuum filtered and rinsed with wash buffer (50 mmol/L Tris-HCl, 5 mmol/L MgCl_2_, 2.5 mmol/L EGTA, and 0.5% fatty acid-free BSA, pH 7.4) on a Filtermate cell harvester through Packard GF/B filters presoaked in 0.3% polyethylenimine.

For competition binding, membrane preparations were incubated with 0.3 nM [^3^H]CP55940 in the presence or absence of increasing concentrations of ligands for 60 min at 30°C in a final volume of 0.2 mL of 50 mmol/L Tris-HCl, 5 mmol/L MgCl_2_, 2.5 mmol/L EGTA, 0.1% fatty acid-free BSA, and 1% DMSO, pH 7.4, buffer, gently shaking. Non-specific binding was defined with 10 μM CP55940. All binding reactions were terminated by vacuum filtration onto 0.5% polyethylenimine presoaked GF/B filter plates (Packard) followed by seven brief washes with 2 mL of ice-cold binding buffer containing 0.5% fatty acid-free BSA. Plates were dried at 50°C for 1 h and liquid scintillation counting was used to determine bound radiolabel. IC_50_ values and Hill slopes were determined by a four parameter logistic model using ActivityBase (ID Business Solution, Guilford, UK). pK_i_ values were determined by the generalized Cheng-Prusoff equation (Yung-Chi and Prusoff, 1973).

## Author contributions

DV and MA performed experiments. CU contributed to binding assay experiments. DV, MA, GB analyzed the data. All the authors contributed toward designing the experiments and writing the manuscript.

### Conflict of interest statement

The authors declare that the research was conducted in the absence of any commercial or financial relationships that could be construed as a potential conflict of interest.
